# Métastase exceptionnelle d'un glioblastome

**DOI:** 10.11604/pamj.2015.20.349.6373

**Published:** 2015-04-10

**Authors:** Mohamed Moukhlissi, Farid Naciri

**Affiliations:** 1 Centre Régionale d'Oncologie, Oujda, Maroc

**Keywords:** Glioblastome, parties molles, métastases, Glioblastoma, soft tissue, metastases

## Abstract

Les métastases extracrâniennes découlant de glioblastome sont rares et le mécanisme de leur diffusion n'est pas bien connu. Nous rapportons le cas d'un homme suivi pour un glioblastome traité par chirurgie suivie d'une radiothérapie encéphalique avec Temozolamide en concomitant, et qui a présenté une métastase des parties molles.

## Introduction

Les glioblastomes sont des tumeurs du système nerveux central hautement invasives, à extension purement locorégionale (limitée au système nerveux central). Les métastases systémiques sont très rares voire exceptionnelles. Nous rapportons un cas d'un patient suivi pour un glioblastome qui présente au cours du traitement adjuvant par Temozolamide une métastase des parties molles confirmée histologiquement.

## Patient et observation

**Figure 1:**
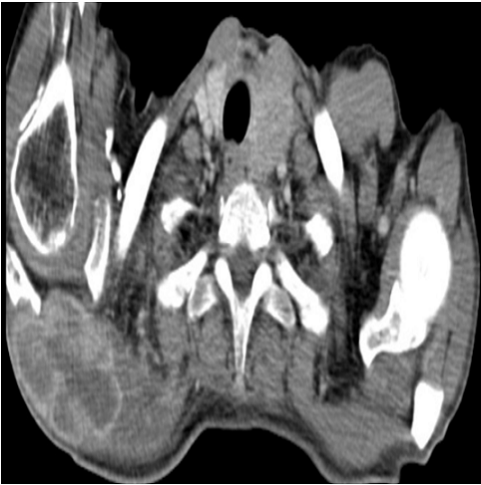
Masse tissulaire pariétale, intéressant les parties molles sus scapulaires droites avec envahissement des muscles deltoïdes et lyse osseuse de l'acromion (parietal tissue mass, involving soft tissue above right scapular with invasion of the deltoid muscle and bone lysis of the acromion)

**Figure 2:**
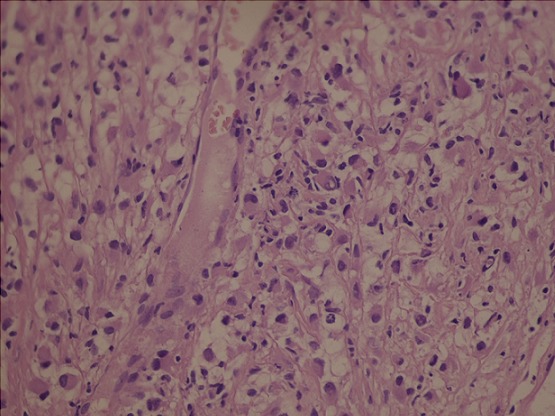
Coloration standard hematoxyline éosine (standard hematoxylin eosin)

**Figure 3:**
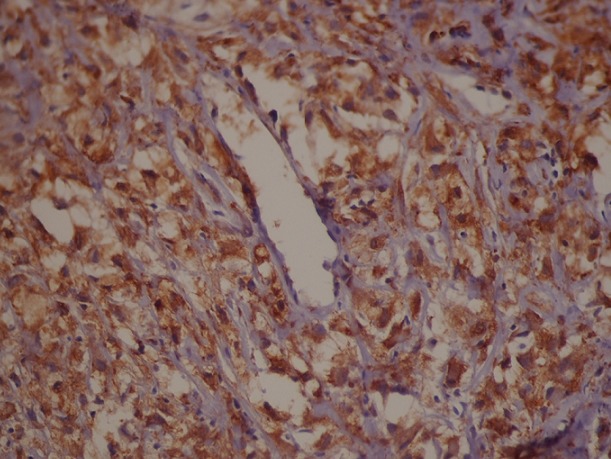
Marquage fort en immunohistochimie de l'anticorps anti- NSE (Strong labeling in an immunohistochemical antibodies NSE)

**Figure 4:**
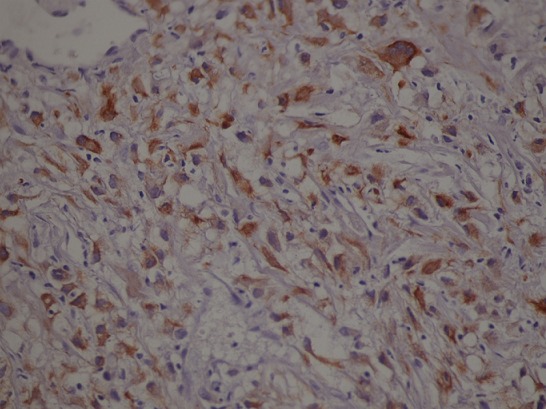
Marquage immunohistochimique de l'anticorps anti- KL1 (immunohistochemical staining of an antibody anti KL1)

Il s'agit d'un patient de 65 ans pris en charge au service de radiothérapie pour une lésion cérébrale pariéto-occipitale droite découverte devant des céphalées persistantes, des vertiges et des épisodes de vomissements. L'exérèse macroscopiquement complète de la masse a permit de poser le diagnostic de GBM. Il fut traité par radiothérapie encéphalique avec Temozolomide concomitant, puis en adjuvant par des cures mensuelles. Six mois après l'exérèse, il présenta des douleurs dorsales associées à une tuméfaction de l’épaule droite sans signes inflammatoires en regard. L'examen clinque trouve une masse douloureuse fixée à l'os. Une TDM de la région scapulaire droite a objectivé une masse tissulaire pariétale de 84 mm de grand axe, intéressant les parties molles sus scapulaires droites avec envahissement des muscles deltoïdes et lyse osseuse de l'acromion (Figure 1, Figure 2). Le bilan a été complété par une TDM thoraco-abdomino-pelvienne qui n'a pas objectivé d'autres lésions, une IRM cérébrale n'a pas montré de signes de progression tumorale. Une biopsie de la masse a été réalisée. L’étude histologique et immuno-histochimique étaient en faveur d'une métastase du glioblastome déjà connu avec une positivité des marqueurs GFAP, NSE et KL 1 (Figure 3, Figure 4). Le traitement a consisté en une reprise de Temozolamide et une radiothérapie sur la masse à la dose de 60 gy. Après un recul de 8 mois on a remarqué une stabilité de la lésion de l’épaule avec disparition de la douleur sans signes de progression tumorale au niveau cérébrale.

## Discussion

Les glioblastomes sont considérés parmi les tumeurs hautement agressives, cependant ce potentiel agressif reste limité au système nerveux central. Les métastases systémiques sont exceptionnelles pouvant être expliquées par l'absence de drainage lymphatique du système nerveux central mais surtout l'incapacité des cellules gliomateuses de franchir la membrane basale des capillaires sanguins [1–3]. En dehors du SNC, les glioblastomes diffusent principalement dans les poumons et les ganglions lymphatiques, mais aussi dans le foie et les os qui représentent 25 à 30% des patients présentant des métastases extracrâniennes [4]. Les mécanismes de dissémination des métastases systémiques des GBM ne sont pas élucidés. La chirurgie pourrait avoir un rôle favorisant, en permettant la pénétration des cellules tumorales dans les vaisseaux sanguins et les lymphatiques extracraniens (exérèse tumorale ou dérivation ventriculo-péritonéale), par rupture de la barrière hémato-encéphalique, bien que quelques cas aient été rapportés en dehors de toute chirurgie ce qui rend aussi valable la théorie d’éventuelles altérations génétiques des cellules gliomateuses leur permettant donc de migrer [5]. La survenue, dans le suivi thérapeutique d'une localisation tumorale systémique, impose une confirmation histologique pour éliminer un deuxième cancer. L’étude immunohistochimique avec des marqueurs tels que la GFAP (glial fibrillary acidic protein) ou Olig2 est hautement contributive [5–7]. Le traitement des métastases systémiques des glioblastomes reste palliatif et repose essentiellement sur la chimiothérapie. L'irradiation palliative peut être un autre choix thérapeutique comme ce fut le cas de notre patient dont la lésion est restée stable après la radiothérapie avec disparition de la douleur [8].

## Conclusion

Les métastases extracrâniennes des glioblastomes sont exceptionnelles, leurs survenues restent encore mal connues malgré les hypothèses avancées dans la littérature, leur pronostic est très mauvais malgré l'arsenal thérapeutique qui existe à l'heure actuelle.
